# The impact of dysmenorrhea and premenstrual syndrome on academic performance of college students, and their willingness to seek help

**DOI:** 10.4274/tjod.galenos.2020.97266

**Published:** 2020-10-02

**Authors:** Esra Bilir, Şule Yıldız, Kayhan Yakın, Barış Ata

**Affiliations:** 1 Koç University School of Medicine, İstanbul, Turkey; 2 Koç University Hospital, Department of Obstetrics and Gynecology, İstanbul, Turkey; 3 Koç University School of Medicine, Department of Obstetrics and Gynecology, İstanbul, Turkey

**Keywords:** Dysmenorrhea, premenstrual syndrome, pelvic pain, survey, academic performance

## Abstract

**Objective:**

To reveal the characteristics and prevalence of dysmenorrhea and Premenstrual syndrome (PMS) among college students and to investigate their impact on their academic performance.

**Materials and Methods:**

This cross-sectional study was conducted between December 2017 and January 2018 at Koç University, Turkey. An online survey that included multiple-choice and short paragraph questions was prepared. Female students aged between 18 and 27 years were invited with an email to provide online informed consent to proceed to the survey.

**Results:**

The final analysis included 352 students. The prevalence of dysmenorrhea was found as 90.1%. Fifty-six percent of the participants reported lower academic performance during menstruation. However, only 32.8% of the students with dysmenorrhea presented to the gynecology clinic. The prevalence of PMS alone and with dysmenorrhea was 71.3% and 65.9%, respectively. The most common symptom among those who reported affected academic performance was depression (prevalence of 27.5%). However, only 19.9% of students with PMS consulted a healthcare professional.

**Conclusion:**

Symptoms of dysmenorrhea and PMS are generally neglected by students. Quality of life can be affected more than estimated. Considering the reluctance to disclose menstrual disorders, health care providers should be aware of them and ask women about their symptoms during routine visits.

**PRECIS:** PMS and dysmenorrhea are ignored by college students. Early education can be an efficient method to increase awareness and prevent delays in diagnosis and management.

## Introduction

Primary dysmenorrhea, defined as recurrent lower abdominal and/or pelvic pain during menstruation affects 45 to 95% of women of reproductive age^(1)^. Primary dysmenorrhea refers to dysmenorrhea in the absence of an organic pathology, and secondary dysmenorrhea can occur due to gynecologic disorders such as endometriosis, adenomyosis or uterine fibroid, as well as others.

Premenstrual syndrome (PMS) presents with at least one affective symptom (irritability, anxiety, confusion, depression, anger outburst, or social withdrawal) and at least one somatic symptom (abdominal bloating, breast tenderness, headache, or swelling of extremities) during the five days prior to menses and presenting in at least three consecutive menstrual cycles^(2)^. Also, within four days of the onset of menses, symptoms should be alleviated. The most severe form of PMS is premenstrual dysphoric disorder, which is defined by the Diagnostic and Statistical Manual of Mental Disorders, Fifth Edition criteria as one or more of the following symptoms; mood swings/sudden sadness/increased sensitivity to rejection, anger/irritability, sense of hopelessness/depressed mood/self-critical thoughts, or tension/anxiety/feeling on edge^(3)^. The prevalence of PMS was reported as 47.8% in a meta-analysis, which called for further studies from different countries (95% confidence interval: 32.6-62.9)^(4)^.

The impact of dysmenorrhea and PMS on the academic performance of female students has not been studied fully. A survey conducted among female high school adolescents revealed that dysmenorrhea alone or together with PMS was associated with school absenteeism^(5)^. Another survey conducted among female university students focused only on dysmenorrhea and reported a negative effect on the quality of life^(6)^.

The primary aim of this study was to reveal the characteristics and prevalence of dysmenorrhea and PMS in a college population. The secondary aim was to investigate their impact on the academic performance of college students.

## Materials and Methods

This cross-sectional study was conducted at Koç University in Istanbul, Turkey, between December 13^th^, 2017, and January 15^th^, 2018. We prepared an online survey that included multiple-choice and short paragraph questions. To standardize the survey, we conducted a literature review from PubMed; afterwards, we prepared the final form of the survey questions regarding our above-mentioned research purposes. This study was approved by Koç University Institutional Review Board (IRB) (2017. 216.IRB3.119). Female students aged between 18 and 27 years were invited by email for participation. Students whose first language was not Turkish were excluded from the study.

The sample size was calculated as 347 via the Australian National Statistical Service Sample Size Calculator Tool. The total number of female students was 3511, and 347 respondents were needed for 95% confidence and 0.05 as the confidence interval, with a standard error and relative standard error of 0.025 and 5.10, respectively.

### Participants/Materials, Setting, Methods

Participants were required to provide online informed consent in order to proceed to the questionnaire. Identification information was not collected.

Regular menstruation was defined as one occurring with 21 to 35 days intervals. Visual analogue scale (VAS) scores from 0 to 10, where 10 represented the most severe form of pain, were used to quantify pain. The diagnosis of PMS was made according to previously defined criteria^(2)^.

### Statistical Analysis

The Statistical Package for the Social Sciences (SPSS) Version 24.0 (Chicago, IL, USA) was used to analyze the data. The statistical analysis was performed with a mean, median, and percentage.

### Questionnaire

The questionnaire comprised three sections. The first section consisted of descriptive questions including age, school, age at menarche, and menstruation characteristics such as regularity and duration. The second section questioned the presence of dysmenorrhea and its characteristics including severity, persistency, duration, accompanying symptoms (nausea, vomiting, headache, migraine, back pain, breast tenderness, decrease in appetite, dizziness, constipation, painful defecation, painful urination, and others), and the respondent’s perception of whether the pain affected their academic performance, annual school absenteeism in days due to dysmenorrhea, whether they disclosed the fact that absence was due to painful menstruation to their instructor, the magnitude of the impact on daily life and social life, if they ever presented to a gynecologist and/or emergency department regarding dysmenorrhea, whether a pelvic ultrasound revealed pathology that would cause dysmenorrhea, their diagnosis for dysmenorrhea, coping mechanisms for the pain (herbs, exercise, rest, hot shower, hot pack, oral/intramuscular/intravenous analgesics, oral contraceptive pills, and intrauterine device), whether they changed their coping mechanisms in the course of time, family history (Fhx) of dysmenorrhea including their mothers, sisters, aunts, and grandmothers, and the diagnosis if Fhx was positive. The third section included questions regarding PMS. The ten PMS symptoms (irritability, anxiety, confusion, depression, angry outburst, social withdrawal, abdominal bloating, breast tenderness, headache, and swelling of extremities) were listed, and the participants were asked to choose the ones they encountered five days prior to their menstrual bleeding. Secondly, they selected the most disturbing symptom among the reported symptoms. Because PMS was described previously as the presence of at least one of the ten symptoms in at least three consecutive menstrual cycles^(2)^, they were asked to choose whether they had the symptoms during more or less than three consecutive menstrual cycles. Whether they consulted a physician about PMS, if yes, their speciality, treatment, and whether they benefited from the treatment were questioned. They were asked to identify the symptom that most affected their academic performance. Annual school absenteeism in days due to PMS and whether or not they disclosed that absence was due to PMS to their instructor were asked. Finally, they were asked to report and quantify the medical conditions they experienced before their period.

## Results

A total of 457 students completed the survey with a participation rate of 13.0%. Only 353 students completed the entire survey. One participant was excluded due to conflicting responses. Hence, the final analysis included 352 students. Demographic features and menstrual cycle characteristics, including menarche, cycle length, duration of bleeding, and regularity of the participants are presented in Table 1.

**Figure 1:**
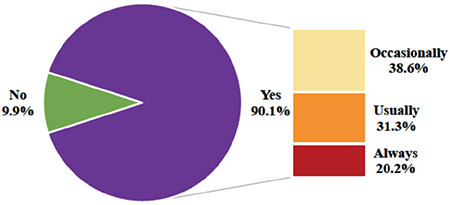
Dysmenorrhea prevalence

The prevalence of dysmenorrhea was found as 90.1%, where the proportion of never, occasionally, usually, and always responses were 9.9%, 38.6%, 31.3%, 20.2%, respectively (Figure 1). The median (25^th^ - 75^th^ percentile) VAS was 8 (7-9). The persistence of dysmenorrhea was 46.3%. The median duration of dysmenorrhea per cycle was 2 (range, 1-6) days. Accompanying symptoms and the coping mechanisms of dysmenorrhea are shown in Table 2. Thirty-one percent of women changed their coping mechanisms over time.

Fifty-six percent of the participants reported lower academic performance during menstruation. The median school absenteeism due to dysmenorrhea was three days annually for 201 people (range, 1-24). Only 12.8% of the students stated that they could disclose that their absence was due to painful menstruation with their instructors without hesitation. Only seven of the nursing and 12 of the medical students shared their menstrual disorders (25.9% and 26%, respectively). Ninety-five percent of the participants reported that dysmenorrhea did not affect their daily life; however, 92% of reported a negative effect on social life. Admission to an outpatient gynecology clinic and the emergency department was 32.8% and 25.8%, respectively. Almost sixty-six percent of the students with dysmenorrhea indicated a positive Fhx for dysmenorrhea. On the other hand, 40% of the students without dysmenorrhea indicated positive Fhx for dysmenorrhea.

According to the aforementioned PMS criteria, we found the prevalence of PMS alone and with dysmenorrhea was 71.3%, and 65.9%, respectively. The distribution of symptoms from most to least common among the students diagnosed with PMS was abdominal bloating 215 (85.7%) and irritability 202 (80.5%), breast tenderness 187 (74.5%), angry outburst 182 (72.5%), anxiety 158 (62.9%), confusion 158 (62.9%), depression 141 (56.2%), social withdrawal 92 (36.7%), headache 74 (29.5%), and swelling of extremities 54 (21.5%) where the participants were allowed to choose more than one option. The most disturbing symptom reported by the students was anger outburst with a prevalence of 25.1% (63 students). Only 19.9% of the students with PMS had consulted a gynecologist, psychiatrist, neurologist, psychologist or endocrinologist (15.5%, 1.6%, 1.2%, 1.2%, and 0.4%, respectively). As treatment among the students with PMS, 7.6% of the participants reported using oral contraceptives. The most common symptom among those who reported affected academic performance was depression with a prevalence of 27.5%. One hundred twenty-two students reported school absenteeism (range, 1-38 days, annually) due to PMS. However, only 11 (4.4%) students stated that they could share that the absence was due to PMS with their instructors without hesitation. Among the nursing and medical students who reported that school absenteeism was due to PMS, 22.2% and 18.2%, respectively, could share with their instructors without hesitation. Finally, the severity of the PMS symptoms is listed in Table 3.

## Discussion

Our survey indicates thatdysmenorrhea is a common problem among university students. More than half of the participants reported that dysmenorrhea affected their academic performance mainly due to absenteeism. Our results also showed that depression was the most common symptom affecting their academic performance, which could perhaps be prevented by consulting a health professional and sharing their problems. Also, more than 70% of students reported experiencing PMS symptoms, and the most common symptom was anger outburst, which affects social life negatively.

Surveys are prone to bias when the response rate is too low. The participation rate in our study was 13%, likely because we inactivated the survey when we reached the calculated sample size. It is possible that students with dysmenorrhea and/or PMS were more likely to complete the questionnaire. This might have caused an overestimation of dysmenorrhea and PMS prevalence. Our study group homogenously consisted of educated women, and the study was anonymous, which gave the confidentiality of identity.

Menstrual disorders such as dysmenorrhea and PMS can affect academic performance, mental well-being, and quality of life. Menstrual disorders are estimated to affect almost 2.5 million women every year^(7)^. The symptoms can be physical, emotional, or behavioral, which is thought to be derived from hormonal fluctuations^(8)^. Even PMS is thought to result from ovulation and hormonal fluctuations and primary dysmenorrhea from increased production of prostaglandins resulting in painful uterine contractions and decreased blood flow; however, the etiology of menstrual disorders is yet to be identified^(8,9)^.

A strong association between quality of life and severity of PMS symptoms has been reported in the literature^(10,11)^. The majority of women in reproductive age have at least one symptom of dysmenorrhea and/or PMS^(12,13,14,15,16)^. We wanted to address the impact of the symptoms of menstrual disorders on the academic performance of female students. Similar to our results, several other studies also reported that menstrual disorders caused school absenteeism, defects in social life, and a decline in academic performance^(17,18,19)^. Absenteeism varied in a range of 18.6% to 80.6% among university students in previous studies^(16,20,21)^.

The retrospective diagnosis of PMS is a limitation of this study. However, because there is not an objective diagnostic criterion of PMS^(22)^, it is difficult to evaluate its accurate prevalence. Further studies should be designed prospectively via providing the participants with the charts for PMS symptoms for at least for three consecutive months. Hence, we would like to ask experts to prepare an up-to-date objective universal guideline.

Intriguingly, highly educated women are hesitant to disclose their menstrual disorders and even their absenteeism being due to dysmenorrhea or/and PMS symptoms to their instructors^(23)^. Even more remarkably, medical and nursing school students, who are expected to have a higher awareness of these disorders, were also reluctant to share the fact that their absenteeism was due to menstrual disorders. Sharing and presenting to a gynecology clinic or for emergency care was also low among the students. This makes one think that most women consider menstrual disorders as natural and do not seek a remedy, rather the try to manage with temporary solutions or to live with it. On the other hand, the lack of seeing a physician due to menstrual disorders also causes a delay in diagnoses for conditions underlying secondary dysmenorrhea, such as endometriosis^(24)^.

Current treatment options for dysmenorrhea include non-hormonal medical therapy such as acetaminophen and non-steroidal anti-inflammatory drugs, hormonal treatment such as contraceptive pills or progestin regimens^(25)^. Complementary and alternative treatment options can be recommended, such as exercise, transcutaneous electrical nerve stimulation, acupuncture and acupressure, behavioral interventions, topical heat, and dietary supplements^(25)^. Most of the respondents improved their coping mechanisms with dysmenorrhea in an unusual way in our study. Some of them reported benefiting from these strategies; however, mostly, they were temporary. Analgesic usage was very common among participants (92.6%). Only 10% of the students were on oral contraceptive pills. Most of the students used alternative methods while managing with their symptoms such as hot packs (63.4%), hot showers (56.6%), rest (34.8%), herbs (14.8%), exercise (9.7%), and hobbies (8.2%).

## Conclusion

Symptoms of dysmenorrhea and PMS are frequently neglected by college students. The symptoms can vary widely among women. Quality of life can be affected more than estimated, and even being a female can be regarded as a misfortune by the affected population. Considering the reluctance to disclose menstrual disorders, healthcare providers should be aware of the fact and ask women about such symptoms in routine visits. Thereby, the symptoms of PMS and dysmenorrhea ignored by women, which affect the quality of life, can be identified, and awareness can be increased. Besides, education for adolescents can be an efficient method to increase awareness and prevent delays in diagnosis.

## Figures and Tables

**Table 1:**
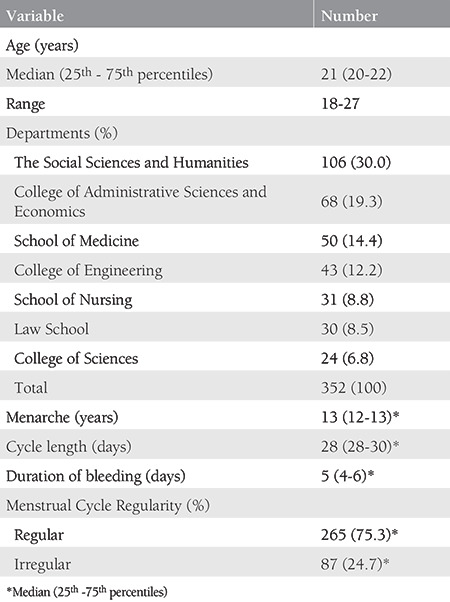
Demographic features of the participants together with menstrual cycle characteristics

**Table 2:**
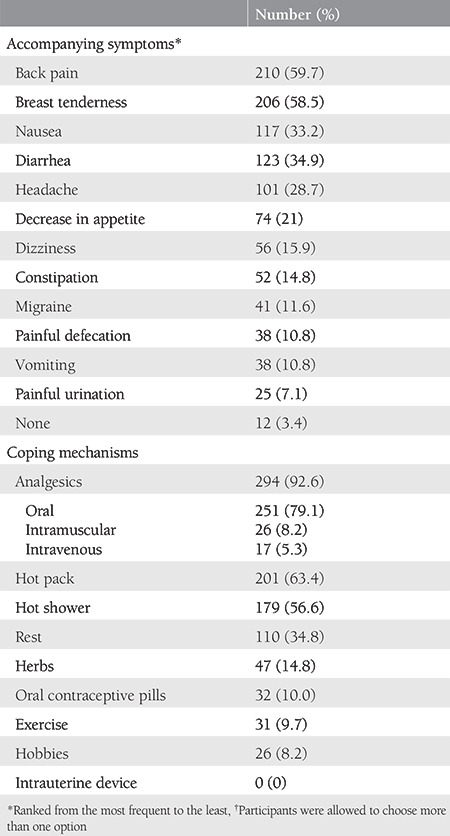
Accompanying symptoms† and coping mechanisms† of dysmenorrhea

**Table 3:**
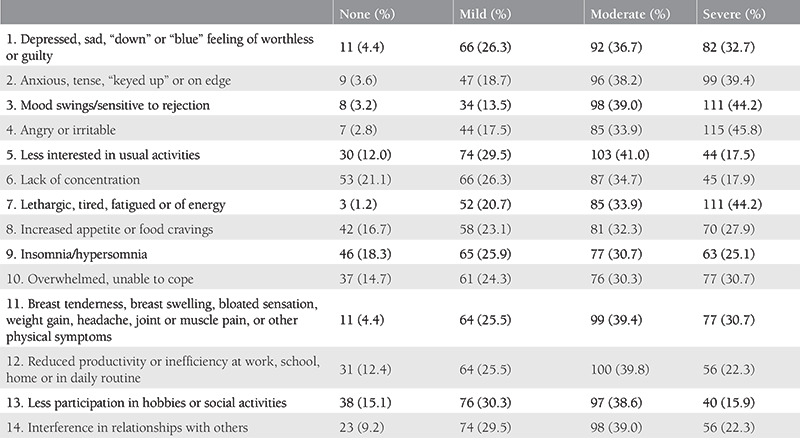
Severity of the premenstrual syndrome symptoms (n=251)

## References

[1] Iacovides S, Avidon I, Baker FC (2015). What we know about primary dysmenorrhea today: a critical review. Hum Reprod Update.

[2] Mortola JF, Girton L, Yen SSC (1989). Depressive episodes in premenstrual syndrome. Am J Obstet Gynecol.

[3] Hantsoo L, Epperson CN (2015). Premenstrual Dysphoric Disorder: Epidemiology and Treatment. Curr Psychiatry Rep.

[4] Direkvand-Moghadam A, Sayehmiri K, Delpisheh A, Kaikhavandi S (2014). Epidemiology of Premenstrual Syndrome (PMS)-A Systematic Review and Meta-Analysis Study. J Clin Diagn Res.

[5] Wilson CA, Keye Jr WR (1989). A survey of adolescent dysmenorrhea and premenstrual symptom frequency. A model program for prevention, detection, and treatment. J Adolesc Health Care.

[6] Unsal A, Ayranci U, Tozun M, Arslan G, Calik E (2010). Prevalence of dysmenorrhea and its effect on quality of life among a group of female university students. Ups J Med Sci.

[7] Clayton AH (2008). Symptoms related to the menstrual cycle: diagnosis, prevalence, and treatment. J Psychiatr Pract.

[8] Bäckström T, Andreen L, Birzniece V, Björn I, Johansson I-M, Nordenstam-Haghjo M, et al (2003). The role of hormones and hormonal treatments in premenstrual syndrome. CNS Drugs.

[9] Sharghi M, Mansurkhani SM, Larky DA, Kooti W, Niksefat M, Firoozbakht M, et al (2019). An update and systematic review on the treatment of primary dysmenorrhea. JBRA Assist Reprod.

[10] Borenstein JE, Dean BB, Endicott J, Wong J, Brown C, Dickerson V, et al (2003). Health and economic impact of the premenstrual syndrome. J Reprod Med.

[11] Hylan TR, Sundell K, Judge R (1999). The impact of premenstrual symptomatology on functioning and treatment-seeking behavior: experience from the United States, United Kingdom, and France. J Womens Health Gend Based Med.

[12] Nisar N, Zehra N, Haider G, Munir AA, Sohoo NA (2008). Frequency, intensity and impact of premenstrual syndrome in medical students. J Coll Physicians Surg Pak.

[13] Balık G, Ustüner I, Kağıtcı M, Sahin FK (2014). Is there a relationship between mood disorders and dysmenorrhea?. J Pediatr Adolesc Gynecol.

[14] Nwankwo TO, Aniebue UU, Aniebue PN (2010). Menstrual disorders in adolescent school girls in Enugu, Nigeria. J Pediatr Adolesc Gynecol.

[15] Pinar G, Colak M, Oksuz E (2011). Premenstrual Syndrome in Turkish college students and its effects on life quality. Sex Reprod Healthc.

[16] Potur DC, Bilgin NC, Komurcu N (2014). Prevalence of dysmenorrhea in university students in Turkey: effect on daily activities and evaluation of different pain management methods. Pain Manag Nurs.

[17] Aziato L, Dedey F, Clegg-Lamptey JN (2014). The experience of dysmenorrhoea among Ghanaian senior high and university students: pain characteristics and effects. Reprod Health.

[18] Iliyasu Z, Galadanci HS, Abubakar IS, Ismail AO, Aliyu MH (2012). Menstrual patterns and gynecologic morbidity among university students in Kano, Nigeria. J Pediatr Adolesc Gynecol.

[19] Agarwal AK, Agarwal A (2010). A study of dysmenorrhea during menstruation in adolescent girls. Indian J Community Med.

[20] Eryilmaz G, Ozdemir F (2009). Evaluation of menstrual pain management approaches by Northeastern Anatolian adolescents. Pain Manag Nurs.

[21] Tangchai K, Titapant V, Boriboonhirunsarn D (2004). Dysmenorrhea in Thai adolescents: prevalence, impact and knowledge of treatment. J Med Assoc Thai.

[22] DeCherney AH, Nathan L, Laufer N, Roman AS (2019.). Premenstrual Syndrome. Current Diagnosis & Treatment: Obstetrics & Gynecology (12th ed). McGraw-Hill Education.

[23] Ballagh SA, Heyl A (2008). Communicating with women about menstrual cycle symptoms. J Reprod Med.

[24] Greene R, Stratton P, Cleary SD, Ballweg ML, Sinaii N (2009). Diagnostic experience among 4,334 women reporting surgically diagnosed endometriosis. Fertil Steril.

[25] Burnett M, Lemyre M (2017). No. 345-Primary Dysmenorrhea Consensus Guideline. J Obstet Gynaecol Can.

